# Exploiting Knowledge on Structure–Activity Relationships for Designing Peptidomimetics of Endogenous Peptides

**DOI:** 10.3390/biomedicines9060651

**Published:** 2021-06-07

**Authors:** Juan J. Perez

**Affiliations:** Department of Chemical Engineering, Universitat Politecnica de Catalunya, 08028 Barcelona, Spain; juan.jesus.perez@upc.edu

**Keywords:** peptidomimetics, endogenous peptides, bradykinin, angiotensin, structure–activity relationships

## Abstract

Endogenous peptides are important mediators in cell communication, being consequently involved in many physiological processes. Their use as therapeutic agents is limited due to their poor pharmacokinetic profile. To circumvent this drawback, alternative diverse molecules based on the stereochemical features that confer their activity can be synthesized, using them as guidance; from peptide surrogates provided with a better pharmacokinetic profile, to small molecule peptidomimetics, through cyclic peptides. The design process requires a competent use of the structure-activity results available on individual peptides. Specifically, it requires synthesis and analysis of the activity of diverse analogs, biophysical information and computational work. In the present work, we show a general framework of the process and show its application to two specific examples: the design of selective AT1 antagonists of angiotensin and the design of selective B2 antagonists of bradykinin.

## 1. Introduction

Endogenous peptides are important mediators of hormonal, paracrine and neuronal cell communication, being consequently involved in the regulation of many physiological processes [1]. They are produced in diverse tissues by the action of proteolytic enzymes on precursor proteins and stored in secretory vesicles for their acute release when needed, after reception of an external stimulus [2]. Actions of endogenous peptides are mediated through G-protein coupled receptors (GPCRs) and receptor tyrosine kinases (RTKs) [3]. The former typically involve short peptides >30 residues, whereas the latter typically involve larger peptides and proteins. Specifically, more than 90 GPCRs are activated by peptides, accounting for ~30% of the receptors in class A and 100% of the receptors in class B1 [4]. Examples include the endorphins, tachykinins, members of the secretin family, ghrelin, somatostatin, oxytocin and vasopressin, endothelins, cholecystokinin, bradykinin, bombesin or angiotensin II, among others. On the other hand, RTKs have peptides or proteins as endogenous ligands including growth factors, cytokines and hormones. The epidermal growth factor or insulin are ligands with the shorter number of residues among the 58 RTKs known [5].

In general, peptides exhibit high activity and specificity to their targets, a wide spectrum of therapeutic action, low levels of toxicity, structural diversity and absence or low levels of accumulation in body tissues. The broad spectrum of signaling activities exerted by endogenous peptides makes them attractive molecules for pharmaceutical intervention. Moreover, they can be used as raw models to design peptide analogs, extending their spectrum of pharmacological actions. Indeed, endogenous peptides exhibit agonistic pharmacodynamic profiles, but small modifications in their sequence permits designing analogs with antagonistic profiles. In addition, diverse non-endogenous peptides have also been used in the past to inhibit specific biological actions such as enzyme inhibition or protein-protein interactions disruption [6].

Despite their advantages, peptides have a low pharmacokinetic profile, exhibiting poor oral bioavailability, low absorption and fast degradation by peptidases [7]. In addition, they also exhibit immunogenicity [8]. Different techniques have been used in the past to overcome these drawbacks, including the design of hydrolysis-resistant analogs, cyclization or new delivery procedures, increasing the number of available peptides used as therapeutic agents [6,9]. Nowadays, more than 80 peptide drugs have reached the market for the treatment of a wide range of diseases [9].

An alternative approach to bypass the poor absorption, distribution, metabolism and excretion (ADME) profile peptides exhibit is through the design of peptidomimetics, small molecules that are optimized, guided by the stereochemical features that confer peptides their activity [10,11]. The peptidomimetic concept was coined about 45 years ago with the discovery of the enkephalins as endogenous ligands of the opioid receptors [12]. At that time, morphine, the active compound extracted from the *Papaver somniferum* and used as a potent analgesic for more than 150 years, was known to be an agonist of the opioid receptors from early sixties [13]. The discovery of the enkephalins prompted to hypothesize that the activity of morphine was due to the spatial distribution of specific chemical moieties of its structure that mimic those of the bound conformation of the endogenous peptides [14]. Therefore, peptidomimetics are small molecules that mimic key stereochemical features of the bioactive conformation of a target peptide. The purpose of this review is to illustrate the process followed in the past to design peptidomimetics guided by the features of the corresponding endogenous peptides, with the help of two examples: angiotensin II AT1 antagonists and bradykinin B2 antagonists.

## 2. Roadmap to Design Peptidomimetics

The roadmap for peptidomimetics design is simpler when the 3D structure of the peptide/receptor complex is available. In this case, direct inspection of the structure permits the identification of key features that characterize peptide/receptor interaction. Then, a specific chemical scaffold can be used to attach key chemical groups in the right spatial distribution, or alternatively, carry out a virtual screening study to find hits that mimic peptide/receptor interaction. This step is then followed by a hit-to-lead optimization process. Successful examples of this approach include, among others, the design of second-generation HIV protease inhibitors such as the cyclic-urea-based inhibitor SD146 [15] or the protein–protein interactions (PPIs) inhibitor venetoclax, a highly selective Bcl-2 inhibitor that was approved by the US FDA in April 2016 as a second-line treatment for chronic lymphocytic leukemia [16].

Several biophysical techniques are available to obtain the 3D structure of the complex. X-ray crystallography and cryogenic electron microscopy are the most widely used techniques [17]. Nuclear Magnetic Resonance (NMR) spectroscopy, both solid and solution, has also been successfully used to obtain 3D structures of complexes, mainly in those cases where crystals have been difficult to grow, although there is a limitation on the size of the systems studied [18]. Furthermore, a great advantage of NMR studies is that structures can be studied in diverse environments including physiological conditions through in-cell NMR techniques [19]. Integrated approaches that include NMR with X-ray crystallography or cryogenic electron microscopy [20] or techniques, such as small-angle X-ray/neutron scattering (SAXS/SANS) [21], chemical cross-linking [22] or mass spectrometry [23], are also used for this purpose.

**Figure 1:**
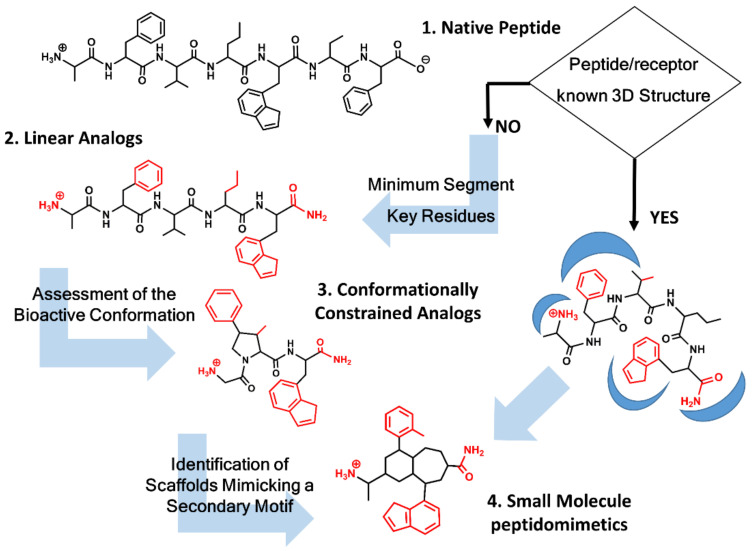
Peptidomimetics design roadmap. Starting from the native peptide sequence (**1**) when the 3D structure of the peptide/receptor complex is not known, first step regards the synthesis of linear analogs (**2**) to establish the shortest peptide fragment with activity and to identify key residues for receptor recognition (in red). Next step consists of designing conformationally constrained analogs (**3**), using information about the secondary structure of the peptide in its bound conformation. The final step is the identification of small molecule scaffolds (**4**) that permit a correct spatial arrangement of relevant chemical groups. Knowledge of the peptide/receptor 3D structure permits to undertake a shortcut.

Unfortunately, in most of the cases, the 3D structure of the peptide/receptor complex is not available, and the design process requires the identification of key residues, as well as information about the geometrical features of the bioactive conformation concomitantly. Figure 1 summarizes the process followed to design a peptidomimetic depending on the structural information available. Knowledge of key residues responsible of the ligand-receptor interaction requires first establishing the shortest peptide segment that retains biological function. Thus, for example, Met-enkephalin is the 5-residue N-terminus segment of the 31-residue peptide β-endorphin retaining full agonist activity toward the opioid receptors [24]. Similarly, the last four residue segments at the C-terminus of cholecystokinin represent the shortest fragment of the native peptide retaining full activity [25]. Next, identification of key residues for receptor recognition can be performed by means of an alanine scan, involving a systematic substitution of each of the residues in the sequence by alanine [26]. Any affinity loss observed underlines the importance of that residue for recognition, although caution should be taken, since the loss of affinity can also be due to conformational changes forced by the alanine substitution that prevents the peptide to attain its bioactive conformation. Synthesis and pharmacological evaluation of diverse analogs, with modifications on the peptide backbone or on the side chains, provide information about the importance of the nature of specific chemical groups that can be translated to specific intermolecular interactions. The permutation of two consecutive residues can also provide information about the relevance of specific side chains for recognition [10,11]. Finally, replacement of standard residues by their D-amino acid counterparts is also an interesting exercise to pursue. Thus, for example, replacement of Arg^8^ in vasopressin for its D-amino acid counterpart permitted the discovery of a vasopressin V2 receptor antagonist with some of its derivatives currently in the clinic [27].

Knowledge of the features of the bioactive conformation is also key in the design process. NMR or Circular Dichroism (CD) spectroscopy studies can provide information about the secondary structure of the peptide in solution, although caution should be taken since the conformation in solution is not necessarily related to the bioactive conformation. Complementarily, computational studies can also help to understand the conformational features of the bioactive conformation. Methods include exploration of the conformational space using diverse techniques such as molecular dynamics or Monte Carlo and docking of flexible ligands onto their receptor [28]. Once a hypothesis about a specific secondary structure motif the peptide adopts is established, the design process is directed toward its falsation by assessing the activity of conformationally constrained analogs [29]. When these analogs match a geometrical features of the bioactive conformation, they increase the free energy of binding by enhancing the availability of the bioactive conformation with the concomitant reduction of the entropic loss penalty upon binding. Constraints can be local or global. Local constraints are made through the incorporation of conformationally constrained amino acids in their sequence [30] or using specific heterocycles such as cyclic lactams [31] that act as dipeptide mimics [11]. A paradigmatic example of a cyclic lactam peptidomimetic is penicillin G, a potent antibiotic extracted from a *Penicillum fungus*. The molecule is a peptidomimetic of the D-alanine-D-alanine dipeptide that acts as inhibitor of the D-alanine carboxypeptidase, preventing the last step involved in the synthesis of the bacterial peptidoglycan cell wall [32]. In contrast to local constraints, cyclic peptides represent globally constrained analogs. They exhibit an increased peptide resistance to proteases degradation, as well as cell permeability, and can also provide information about the stereochemical features of the bioactive conformation of a linear peptide [33]. For example, in a series of potent cyclic peptides incorporating the binding epitope that mediates cell attachment in membrane-bound integrins—the segment Arg-Gly-Asp (RGD) [34]—it was shown that control of the RGD segment conformation in the ring permits the design selective ligands for different integrins [34,35].

## 3. Example 1. Design of Angiotensin II AT1 Antagonists

Approved in the middle nineties, eight angiotensin blockers are nowadays commercially available for the treatment of hypertension. Collectively known as sartans, its members include losartan, valsartan, irbesartan, azilsartan, candesartan, telmisartan, eprosartan and olmesartan [36]. Their design process represents one of the first examples where the use of computational tools helped to speed-up the hit-to-lead optimization process.

Angiotensin II (AII) forms part of the renin-angiotensin system designed to regulate blood pressure and fluid homeostasis. AII is produced from angiotensin I by the action of the angiotensin-converting enzyme (ACE) stimulating the secretion of aldosterone from the adrenal cortex that in turn increases sodium reabsorption in kidneys. The peptide also exhibits a potent vasoconstrictor profile that helps to elevate blood pressure [37]. AII is an octapeptide hormone of sequence Asp^1^-Arg^2^-Val^3^-Tyr^4^-Ile^5^-His^6^-Pro^7^-Phe^8^ that mediates its actions through the AT1 and AT2 receptors, members of the GPCR superfamily [37]. Activation of the AT1 receptor initiates signaling pathways that participate in growth and remodeling of the human vascular system including vasoconstriction, sodium and water retention and change in myocyte growth. Chronic stimulation causes cardiac remodeling in the heart, which results in left ventricular hypertrophy, dilation and dysfunction, eventually leading to heart failure. On the other hand, activation of the AT2 receptor fine-tunes the regulation of natriuresis, body temperature, blood pressure, reproduction, embryonic development, cell differentiation, tissue repair and apoptosis. AT2 receptors are upregulated in pathophysiological processes such as cardiac remodeling following hypertension and myocardial infarction, heart failure and stroke [37]. Taking into account the success of ACE inhibitors for the treatment of hypertension, interest in designing small molecule AT1 antagonists began in the late sixties as alternative drugs for therapeutical intervention [38].

Because, in the early seventies, there was no experimental structure of the AII bound to the AT1 receptor available, the design process involved structure–activity studies of AII analogs. After the discovery of AII, it was early established that Val^3^-Tyr^4^-Ile^5^-His^6^-Pro^7^-Phe^8^ is the shortest segment required for full intrinsic activity, although it is also known that the guanidine group of Arg^2^ plays an important role for binding. Furthermore, the Ala-scan revealed that substitution in positions 4 and 6 reduces drastically the affinity of the analogs. Interestingly, the Ala substitution in position 8 turns the analog into an antagonist. It was also found that the hydroxyl group of Tyr^4^ is necessary for receptor activation. These studies led to the discovery of salarasin [Sar^1^,Leu^8^]-AII (where Sar stands for sarcosine), one of the most potent linear peptide AT1 antagonists described [39]. The compound was used as a proof of concept to demonstrate the role of AII antagonists in reducing blood pressure in vivo [40]. It was only later demonstrated that the compound is a partial agonist of the AT1 receptor [41].

Concerning the features of the bioactive conformation of AII, diverse experimental and modeling studies suggested that the peptide adopts an inverse γ-turn involving residues 3–5 [42]. Subsequent NMR studies confirmed this hypothesis, pointing that the three aromatic rings of the peptide cluster together in non-aqueous media [43]. To confirm this hypothesis, a series of cyclic analogs constrained by a disulfide bridge between positions 3 and 5 were synthesized. Specifically, cysteine or homocysteine was used in position 3; whereas cysteine or *trans*-4-mercaptoproline was used in position 5. Analogs such as cyclo[Sar^1^,Hcys^3,5^]-AII or cyclo[Sar^1^,Cys^3^,Hcy^5^]-AII [44], and later, the cyclo[Sar^1^,Hcy^3^,MPt^5^]-AII [45], were among the most potent cyclic analogs synthesized with affinities similar to those of the native peptide. Geometrical comparison of sets of low-energy conformers of AII and the analogs [MetPhe^4^]-AII and [Pro^5^]-AII permitted to propose a model for the receptor-bound conformation compatible with the conformational features of the cyclic AII analogs described above. This model permitted to define a four-point pharmacophore for the AT1 receptor binding, including the three aromatic moieties of residues Tyr^4^, His^6^ and Phe^8^ together with the C-terminal carboxyl group [46]. Using this pharmacophore, the same authors also designed the analog [D-Tyr^4^, Pro^5^]-AII, which exhibits low nanomolar affinity toward the AT1 receptor.

**Figure 2:**
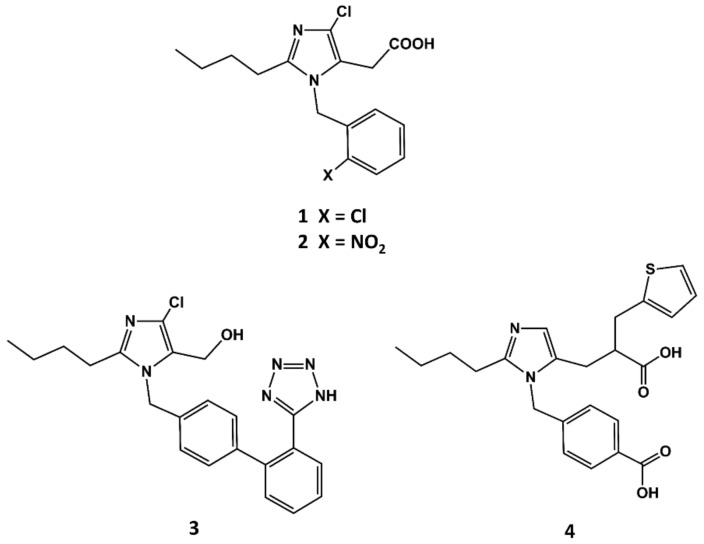
Chemical structures of S-8307 (**1**), S-8308 (**2**), losartan (**3**) and eprosartan (**4**).

The first small molecule peptidomimetic AT1 antagonist was disclosed in the early 1990s, before a solid hypothesis of the conformational features of the bioactive form of AII was built. This was due to the breakthrough discovery of the 1-benzylimidazol-5-acetic acid derivatives such as S-8307 (**1** in Figure 2) and S-8308 (**2** in Figure 2) as weak and selective AII AT1 antagonists from a screening program of bacterial broths [47]. Starting from the hypothesis that compounds S-8307 and S-8308 mimic the C-terminal segment of AII when bound to the receptor, scientists at DuPont discovered losartan (**3** in Figure 2) and scientists at SKB discovered eprosartan, independently (**4** in Figure 2).

**Figure 3:**
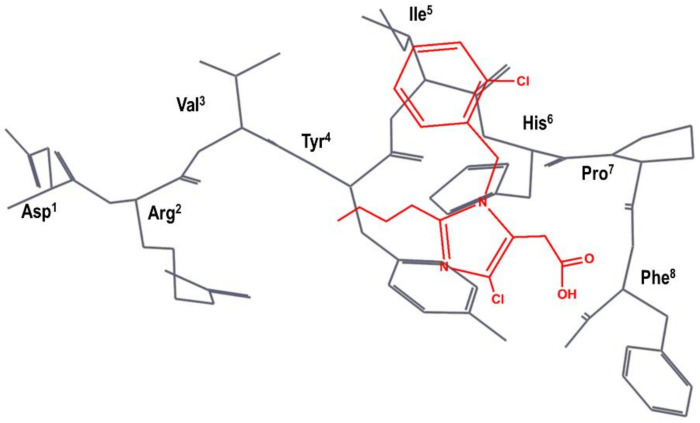
DuPont hypothesis of the superimposition of S-8307 (in red) to the AII bioactive conformation (in black), as described in reference [49]. The Figure was re-constructed using the bioactive conformation of AII taken from pdb id: 6os0.

The team at DuPont superimposed S-8307 to a model of the bioactive conformation of AII previously reported [48], in such a way that the carboxyl group was aligned with the C-terminal carboxyl group of AII; the imidazole nitrogens with those of the histidine residue of AII and the benzyl group were pointed toward the N-terminus of the peptide, as can be seen schematically in Figure 3 [49]. Subsequently, they considered the benzyl group of S-8307 as the most suitable moiety for a systematic extension of the molecule toward the N-terminus of AII. Indeed, a carboxylic acid group in *para* of the phenyl ring was introduced to mimic the Tyr^4^ side chain with a 10-fold increase binding affinity. After diverse substitutions, a second phenyl group was added to culminate the process with the discovery of losartan (**2** in Figure 2), the first AII peptidomimetic commercially available [49]. Losartan is metabolized, in many animal species as well as humans, to EXP3174, a losartan analog with the hydroxymethyl group of position 5 of the imidazole ring oxidized to a carboxylic acid, giving rise to a more potent antagonist that has been used as raw model for the development of other AII antagonists.

**Figure 4:**
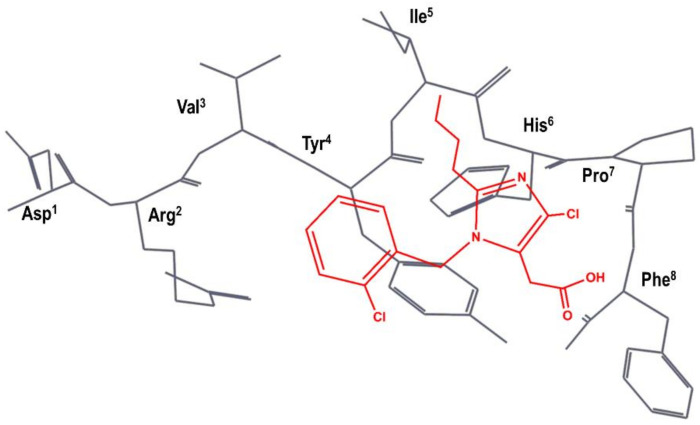
SKB hypothesis of the superimposition of S-8307 (in red) to the AII bioactive conformation (in black), as described in reference [50]. The Figure has been re-constructed using the bioactive conformation of AII taken from pdb id: 6os0.

A team of researchers at SKB independently carried out a different superimposition of S-8307 onto AII, as shown schematically in Figure 4. Specifically, the N-benzyl group of S-8307 was superimposed to the Tyr^4^ aromatic side chain and the carboxyl moiety superimposed to the Phe^8^ carboxyl group of AII, with the 2-butyl moiety lying in the hydrophobic region near Ile^5^. Accordingly, they considered that the imidazole ring was a suitable scaffold for the positioning of pendant groups to get a better overlay with the peptide. In the first modifications of the lead, the authors rigidified the carboxyl moiety to produce an imidazole-5-acrylic acid derivative to which they added a α-benzyl group to the acrylic acid side chain in order to mimic Phe^8^ side chain. This process produced a compound that is fifteen times more potent than S-8307. After several modifications, in order to mimic more closely the side chain of Tyr^4^, the chlorine of the N-benzyl group is removed, and a carboxyl moiety is added in *trans* to produce eprosartan (**4** in Figure 2) [50].

**Figure 5:**
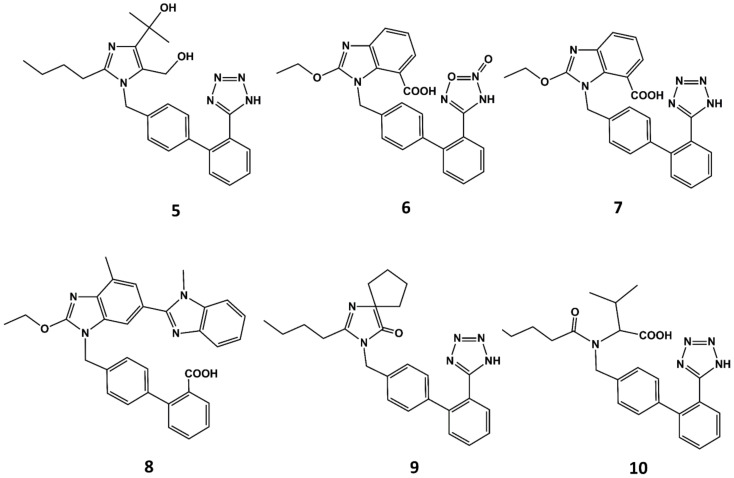
Chemical structures of diverse sartans: olmesartan (**5**), azilsartan (**6**), candesartan (**7**), telmisartan (**8**), irbesartan (**9**) and valsartan (**10**).

With the exception of eprosartan, the rest of the angiotensin blockers commercially available can be considered derivatives of losartan or its metabolite EXP3174 [51]. These compounds are more potent than the parent compound and exhibit improved ADME profiles [52]. Apart from eprosartan, these compounds share common structural features: an ortho substituted biphenyl ring with an acidic moiety and a single or fused heterocyclic or even acyclic part in which a short alkyl chain with 3 or 4 carbon atoms is attached. Thus, for example olmesartan (**5** in Figure 5) is an analog of EXP3174 with the butyl chain replaced by a propyl chain and the chloride atom replaced by a terbutanol group. Inspired by the variety of functional groups tolerated at positions C4 and C5 of the imidazole ring in losartan analogs, it was thought feasible to join both carbons to produce ring-fused imidazoles. Azilsartan (**6** in Figure 5), candesartan (**7** in Figure 5) and telmisartan (**8** in Figure 5) belong to this category of analogs. A different way to attach a ring in positions C4 and C5 to the imidazole ring is shown in irbesartan (**9** in Figure 5). This 4-spirocyclopentaneimidazolin-5-one uses a carbonyl group as hydrogen bond acceptor in place of the C5 hydroxymethyl group of losartan. Finally, valsartan (**10** in Figure 5) is a potent AII antagonist where the imidazole ring is replaced by an acylated amino acid [53].

**Figure 6:**
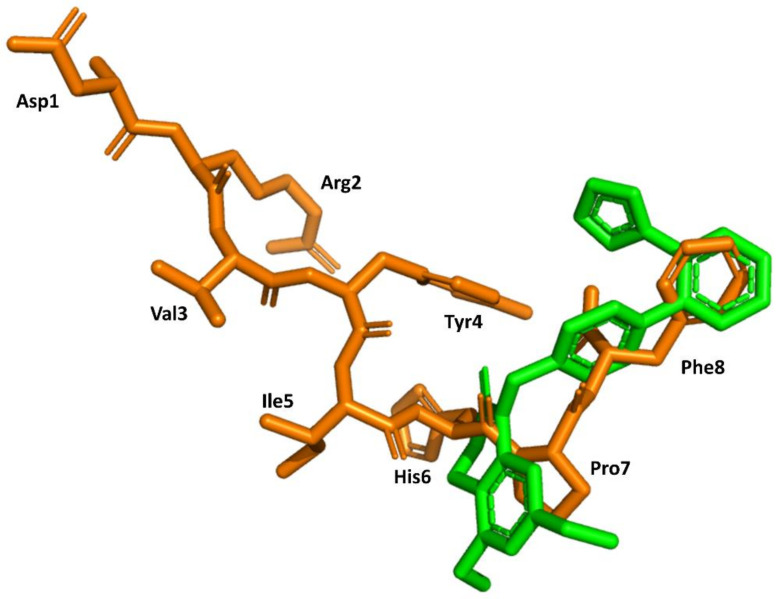
Superimposition of the 3D structure of AII bound to the AT1 receptor (in orange) (pdb id: 6os0) and ZD7155 bound to the AT1 receptor (in green) (pdb id: 4yay).

Structural knowledge about the angiotensin receptors has improved dramatically in recent years. The crystallographic structures of the AT1 receptor in complex with AII antagonists, such as ZD7155 (pdb id: 4YAY) [54] and olmesartan (pdb id: 4ZUD) [55], have recently been reported. Moreover, a structure of the complex AT1 in its active state with AII has also recently been reported (pdb id: 6OS0) [56]. This information can be used to trace back the success of the discovery process described above. Specifically, the structure of AII bound to the AT1 receptor shows its C-terminus buried deep in the orthosteric binding pocket, whereas the N terminus is facing the solvent through a narrow opening at the extracellular face of the receptor, consistent with previous findings that (3–8)AII preserves full activity. Moreover, it is also consistent with the role of Phe^8^ in activation and explains that its substitution by other hydrophobic amino acids converts the diverse analogs in partial agonists. Moreover, the peptide exhibits hydrophobic residues Val^3^ and Ile^4^ facing the same side of the peptide forced by an inverse γ-turn centered at Tyr^4^, as predicted previously [42]. Interestingly, the peptide also adopts a cycle mediated by a hydrogen bond between the Tyr^4^ side chain and the carboxyl C-terminal group, pointing to the importance of these two moieties for AII binding. Finally, a comparison of the bound conformations of AII and ZD7155 confirms that sartans’ structures overlay the C-terminus of AII (Figure 6), confirming the initial hypothesis used for the development of sartans in the early nineties [49,50]. Although, it is not clear that any of the initial designing hypotheses used was better than the other.

Presently available angiotensin blockers, despite being quite satisfactory for the treatment of hypertension, exhibit negative inotropism and cardiomyocyte viability, leading to heart failure progression [57]. The knowledge available on the AT1 receptor nowadays can help to reduce their side effects. Indeed, sartans cause a reduction in arterial mean pressure by blockage of the G protein-dependent pathway of the AT1 receptor; however, biased ligands binding the AT1 receptor can be designed to activate parallel or even distinct signaling pathways that may be exploited to reduce the side effects of available drugs [58,59]. The wealth of structural information available can significantly help in developing novel drugs in this direction [60].

## 4. Example 2. Design of Bradykinin B2 Antagonists

Kinins are an essential component of the kallikrein/kinin system designed to counterbalance the renin-angiotensin system involved jointly in the regulation of intravascular volume, blood pressure and tissue repair via inflammatory and proliferative mechanisms. Specifically, kinins are a group of peptides produced by the action of kallikreins on circulating kininogens in response to trauma, inflammation, shock, burns, allergy and some cardiovascular diseases, provoking changes in blood pressure and vasodilatation, increased vascular permeability, stimulation of sensory neurons, vascular and bronchial smooth muscle contraction, intestinal ion secretion, release of prostaglandins and cytokines and the production of nitric oxide [61,62,63]. Recently, bradykinin has been postulated as the trigger of the inflammatory response observed in severe covid-19 patients [64].

Members of this group of peptides include bradykinin (BK) (Arg^1^-Pro^2^-Pro^3^-Gly^4^-Phe^5^-Ser^6^-Pro^7^-Phe^8^-Arg^9^) and its closely related kallidin (Lys^0^-BK), together with their C-terminal metabolites, desArg^9^-BK and Lys^0^-desArg^9^-BK. Their pharmacological actions are mediated by at least two GPCRs, known as B1 and B2. The former is upregulated during inflammation episodes and tissue trauma, whereas the latter is constitutively expressed in a variety of cell types. Members of the kinin family exhibit a differential affinity profile for the two receptors. Whereas BK and Lys^0^-BK exhibit much higher affinity to the B2 receptor, the desArg^9^ metabolites bind only to the B1 receptor, being Lys^0^-desArg^9^, a potent B1 agonist. Due to their role in mediating pain and inflammation, there has been a remarkable interest in the past to design potent kinin antagonists for therapeutical intervention [65].

The discovery of the antagonistic profile of the [D-Phe^7^]BK analog in the late 1980s [66] led to the characterization of the first-generation B2 antagonists including D-Arg-[Hyp^3^,Thi^5,8^,D-Phe^7^]-BK (NPC349) (Hyp = hydroxyproline; Thi = thienylalanine); D-Arg-[Hyp^3^, D-Phe^7^]-BK (NPC567); or D-Arg-[Hyp^3^,D-Phe^7^,Leu^8^]-BK [67]. Despite the fact that these compounds were useful to understand the involvement of BK in diverse pathophysiological processes, they exhibit diverse drawbacks that prevent their direct therapeutical use. Specifically, they exhibit low affinity for the B2 receptor compared with BK itself and are not selective, despite exhibiting a higher affinity for B2 than for B1. Interestingly, the removal of their C-terminal arginine by carboxypeptidases results in a decrease in affinity for the B2 receptor, turning them into selective B1 antagonists.

**Figure 7:**
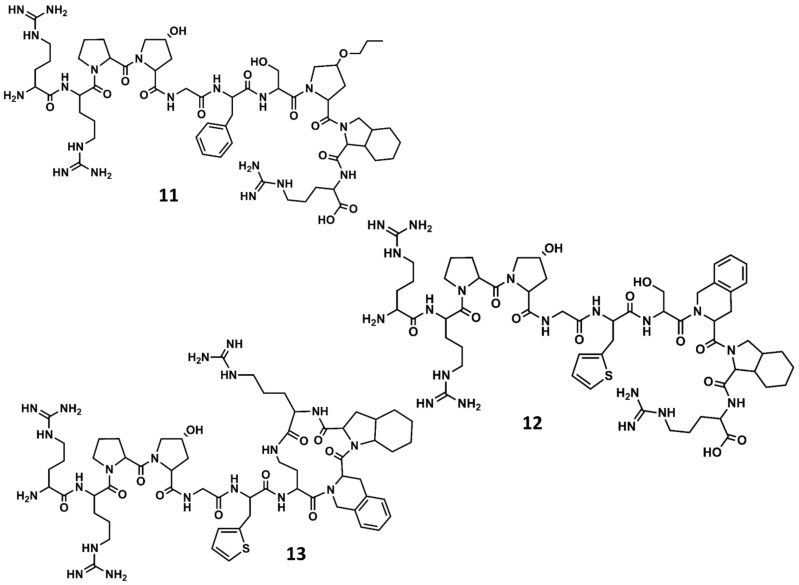
Chemical structures of diverse conformationally constrained B2 antagonists: NPC17731 (**11**); icatibant (**12**) and MEN11270 (**13**).

A second generation of antagonists with improved pharmacological profile was designed on the basis that the C-terminus of BK in solution adopts a β-turn when bound to the receptor, as had been suggested from spectroscopic and molecular modeling studies [68]. Accordingly, diverse analogs incorporating conformationally constrained unnatural amino acids were designed and synthetized, aimed at mimicking the secondary structural motif of BK at the C-terminus. These studies resulted in the discovery of several potent antagonists, including the NPC17731 (D-Arg^0^-[Hyp^3^, D-HypE(*trans*-proyl)^7^, Oic^8^]-BK) (**11** in Figure 7) [69] and the commercially available icatibant (HOE-140) D-Arg^0^-[Hyp^3^,Thi^5^,D-Tic^7^,Oic^8^]-BK (Tic = tetrahydroisoquinoline; Oic = octahydroindole carboxylic acid) (**12** in Figure 7) [70]. Several experimental and computational studies were carried out to support the hypothesis that HOE-140 adopts a β-turn conformation at its C-terminus [71,72,73].

**Figure 8:**
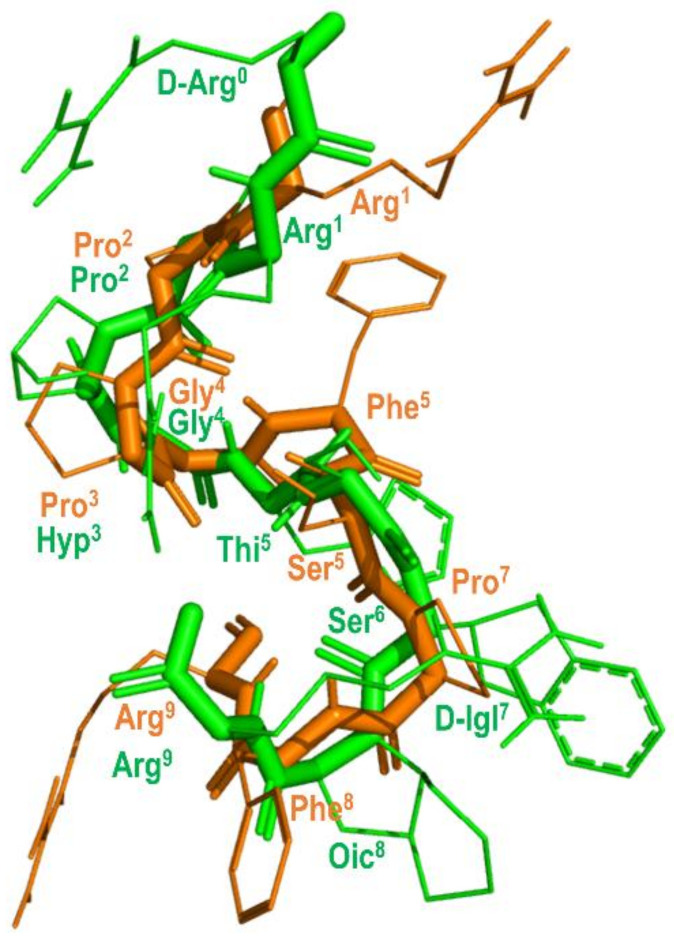
Overlay of the 3D structure of BK bound to the B2 receptor (in orange) (pdb id: 6f3v) and the structure of B-9340 in aqueous solution (in green) (pdb id: 1bdk).

The prediction that BK attains a β-turn when bound to the B2 receptor was recently confirmed from solid-state NMR (ssNMR) experiments [74]. Figure 8 shows the structure of BK bound to the B2 receptor derived from ssNMR studies superimposed with the structure of B-9340 (D-Arg^0^-Arg^1^-Pro^2^-Hyp^3^-Gly^4^-Thi^5^-Ser^6^-D-Igl^7^-Oic^8^-Arg^9^) (Igl = 2-indanylglycine) in aqueous solution, a close peptide analog to HOE-140. As can be seen, the two peptides show an overall good qualitative structural superimposition [75].

In a parallel effort, the search for the shortest peptide sequence retaining antagonistic activity led to the conclusion that the adoption of a β-turn conformation at the C-terminus is a necessary condition for high affinity to the B2 receptor, but not sufficient. In fact, compounds such as the cyclo[Gly-Thi-D-Tic-Oic-Arg] [76] or cyclo[Pro-Orn-D-Tic-Oic-Arg] [77], inspired by the C-terminus of icatibant, show poor affinity for the B2 receptor. Accordingly, the affinity of icatibant and analogs was rationalized in terms of the interactions of the compound with the receptor, such that the β-turn at the C-terminus was thought to occupy a hydrophobic region on the orthosteric pocket, whereas the N-terminal arginine was thought to interact with the negatively charged residues Asp^266^ and Asp^284^ putatively located at the mouth of the receptor [78]. As an indirect proof of concept, the high affinity peptide D-Arg^0^-Arg^1^-Pro^2^-Hyp^3^-Gly^4^-Thi^5^-cyclo[Dab^6^-D-Tic^7^-Oic^8^-Arg^9^] (Dab = diaminobutyric acid) (MEN11270) (**13** in Figure 7) exhibits a cyclic structure at the C-terminus mimicking the β-turn secondary structure and preserves the N-terminal segment of icatibant [79].

The second generation of B2 antagonists corresponds to a set of high-affinity B2 antagonist, highly selective with an improved pharmacokinetic profile, due to their higher resistance to enzymatic degradation. Fruit of these studies’ icatibant (**12** in Figure 7) is currently in the market for the symptomatic treatment of acute attacks of hereditary angioedema in adults with C1-esterase-inhibitor deficiency, which needs to be administered via subcutaneous injection [80]. These compounds exhibit a limited oral bioavailability and require parenteral administration.

**Figure 9:**
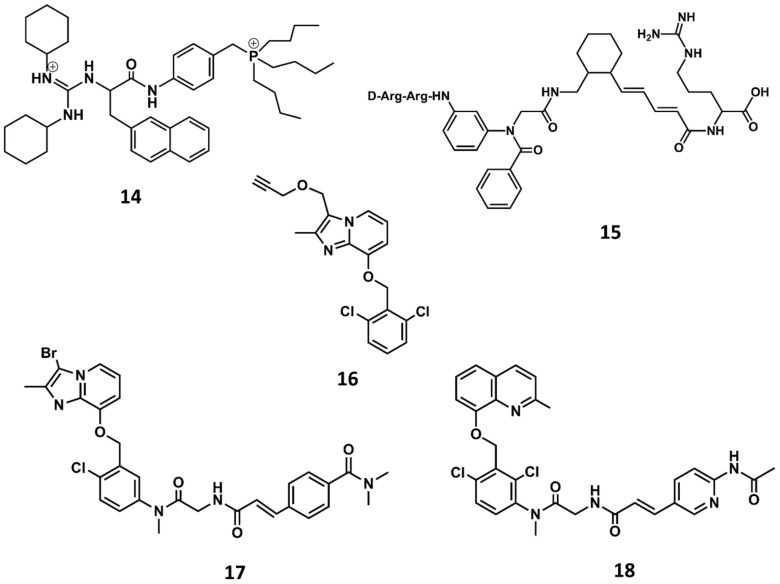
Chemical structures of diverse small molecule B2 antagonists: WN64338 (**14**); NPC-18884 (**15**); Fusijawa hit (**16**); FR167344 (**17**); FR173657 (**18**).

In order to improve oral bioavailability of bradykinin antagonists, research efforts were put forward to design non-peptide B2 selective antagonists. The third generation of B2 antagonists includes diverse molecules disclosed during the 90s and the beginning of the 21st century [81,82]. WIN64338 was the first non-peptide antagonist disclosed (**14** in Figure 9) [83]. It is a small molecule competitive B2 antagonist as demonstrated in diverse in vitro assays. The compound was discovered in a virtual screening using a sketchy hypothesis of the BK bioactive conformation, consisting in two positive charges separated by a hydrophobic spacer to mimic Arg^0^ and Arg^9^ in its bioactive conformation. From an initial hit, there was a subsequent hit-to-lead optimization that permitted the discovery of WIN64338.

A few years later, scientists at Scios Nova, based on the importance of the terminal arginines for binding to the B2 receptor, carried out a combinatorial chemistry approach aimed at identifying scaffolds mimicking the molecular features of peptides with the sequence D-Arg-Arg-X-Y-Arg. Specifically, they considered the synthesis of a series of compounds where organic scaffolds occupied the positions of residues X and Y. The scaffolds studied included different carbocyclic and heterocyclic systems such as the 4-keto-1,3,8-triazaspiro [4,5]decan-3-alkanoic acids, β- and γ-carbolines, phenanthridinones as well as ‘ring opened’ phenanthridinones, linear aminoalkanoic acids and cinnamic acids. The results of this investigation permitted the discovery of the potent B2 antagonist NPC-18884 (**15** in Figure 9) [84].

A breakthrough in the search for non-peptide B2 antagonists came from the discovery of a small molecule lead from a directed screening process carried out using the Fusijawa proprietary database [85]. Specifically, based on the connection of bradykinin and angiotensin II in regulating cardiovascular homeostasis, the screening process was biased to find structures with angiotensin II AT1 antagonistic profile. This study led to the identification of a hit with a dichloro-benzyloxy-imidazopyridine structure (**16** in Figure 9) that exhibited a micromolar B2 antagonistic profile. The addition of a segment to mimic the N-terminus of the peptide improved the affinity of the hit, leading to the discovery of FR167344 (**17** in Figure 9). Further medicinal work found the methylquinoline skeleton to be an adequate replacement for the imidazopyridine moiety, leading to the discovery of the potent B2 antagonist FR173657 (**18** in Figure 9) [85,86].

**Figure 10:**
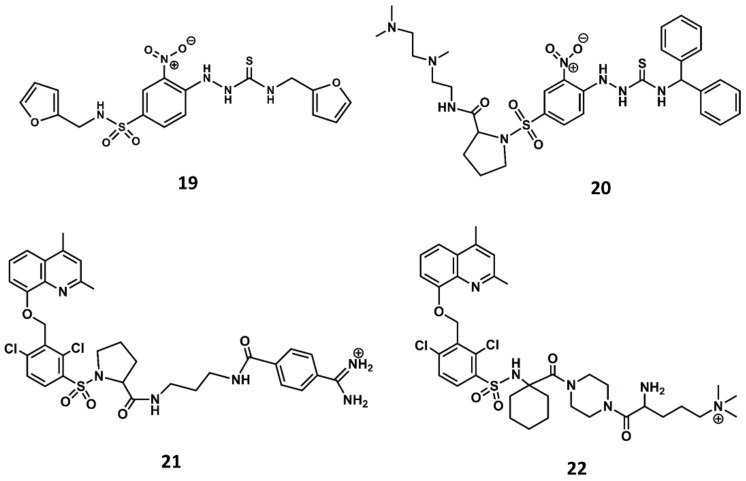
Chemical structures of diverse small molecule B2 antagonists: Novartis hit (**19**); Bradyzide (**20**); anatibant (**21**); fasitibant (**22**).

Similarly, a screening program carried out at Novartis permitted to identify a thiosemicarbazide derivative (**19** in Figure 10) as a hit with micromolar antagonistic profile toward the B2 receptor [87]. Subsequent hit-to-lead optimization led to the discovery of the potent B2 antagonist bradyzide (**20** in Figure 10) [88].

Comparison of the structures of the Fujisawa compounds and bradyzide lead to the discovery of anatibant (**21** in Figure 10) [89] and fasitibant (**22** in Figure 10) [90], after the replacement of the N-methylamide moiety by a sulfonamide linker [89,90]. These compounds exhibit high affinity for the B2 receptor and are selective antagonists. The drawback of these compounds are their limited oral bioavailability due to their high molecular mass, ranging between 500 and 600. Despite these drawbacks, both compounds reached the clinic administered parenterally. The former was considered for the treatment of traumatic brain injury [91] and the latter for the treatment of osteoarthritis [92]. Unfortunately, both compounds were discontinued due to lack of efficacy.

**Figure 11:**
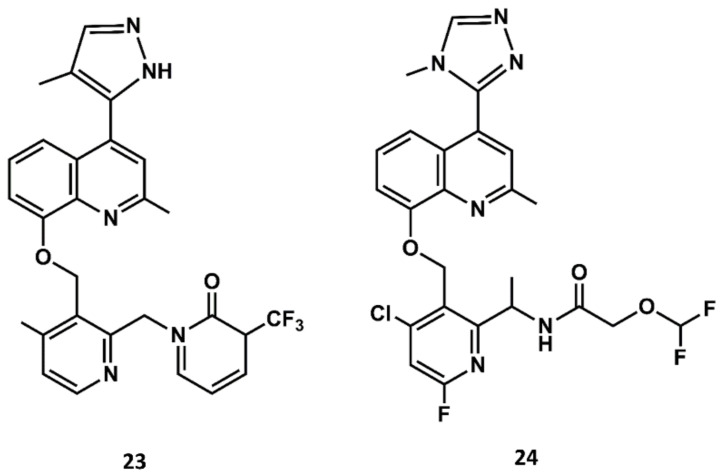
Chemical structures of diverse small molecule B2 antagonists: JSM10292 (**23**) and Compound 3 (**24**).

Aimed at finding compounds with lower molecular mass and retaining the features of these compounds, scientists at Jerini carried out a medicinal chemistry optimization process using the 8-benzyloxy-2-methyl-quinoline moiety, the common scaffold found in FR173657, anatibant and fasitibant, as the starting structure. Docking studies of these ligands onto the B2 receptor constructed by homology modeling suggest that this moiety fits well in a hydrophobic pocket in B2, formed by Leu^114^, Leu^201^, Trp^256^ and Phe^259^, which are not found in the B1 receptor [93,94,95]. The optimization process led to the design and synthesis of JSM10292 (**23** in Figure 11), a potent B2 antagonist with an affinity and a selectivity similar to previous small molecule antagonists disclosed, but with lower molecular mass [96]. This compound, devoid of the long extension attached either to the N-methylamide moiety or the sulfonamide linker, represents a different structure from its predecessors. In a more recent follow up, Pharvaris Netherlands, inspired by the structure of JSM10292, carried out a discovery process that ended up with a novel oral bioavailable B2 antagonist (**24** in Figure 11) with picomolar potency, a high specificity for the human B2 receptor with an improved ADME profile compared with the previous antagonists disclosed [97].

The recent hypothesis published on the role of bradykinin as trigger of the cytokine storm observed in severe COVID-19 patients [64] prompted the use of BK antagonists as therapeutic agents for its treatment. Due to the scarcity of BK antagonists in the market, attention was then turned to drug repurposing as a procedure to identify bradykinin inhibitors in a short time [98]. These investigations led to the discovery that raloxifene is a weak partial agonist of the B2 receptor [99]. Clinical trials are currently been carried out to test the efficacy of icatibant [100] and raloxifene [101] to be used as therapeutic agents for the treatment of COVID-19.

## 5. Conclusions

In this study, we showed, through two examples, how endogenous peptides can be used to guide the design of small molecule drugs. Moreover, it is important to stress that either in the form of peptide surrogate, cyclic analogs or peptidomimetics, the process of designing molecules inspired by native peptides represents a clear example of exploiting structure–activity relationships. Structure–activity studies are key to developing analogs with different pharmacological profiles of the native peptides, turning them from agonists into antagonists. Finally, with the examples included in this report, we like to stress the importance of a combined use of synthetic, biophysical and computational methods to accumulate a solid structure–activity knowledge that permits the development of novel candidates.

In addition to endogenous peptides, enzyme substrates or protein epitopes make peptides a source of inspiration for designing novel drugs [6]. There are numerous examples of drugs derived from peptides in these categories. Thus, peptidase inhibitors belong to a group of drugs successfully derived from peptides such as the ACE inhibitors, e.g., captopril [102] or the HIV protease inhibitors, e.g., saquinavir [103]. In this case, the strategy consists of designing analogs by the substitution of critical peptide bonds with isosteres that make them resistant to proteolytic cleavage while providing certain rigidity [10,11]. Protein–protein interactions inhibitors, such as the Bcl-2 inhibitor venetoclax [16], constitute another group of peptide drug. In both cases, designing small molecules is in principle easier than in the case of GPCR peptide ligands, due to the availability of the 3D structures of the peptide/substrate. Fortunately, advances in crystallography of GPCRs in the last years have led to a better understanding of the hypothesis made on the features of the bound conformation of endogenous peptides [104]. Accordingly, we expect that the development of endogenous peptide peptidomimetics will experience a new dawn in the coming years.

## Data Availability

Not applicable.
